# Convergent Sets of Data from *In Vivo* and *In Vitro* Methods Point to an Active Role of Hsp60 in Chronic Obstructive Pulmonary Disease Pathogenesis

**DOI:** 10.1371/journal.pone.0028200

**Published:** 2011-11-28

**Authors:** Francesco Cappello, Gaetano Caramori, Claudia Campanella, Chiara Vicari, Isabella Gnemmi, Andrea Zanini, Antonio Spanevello, Armando Capelli, Giampiero La Rocca, Rita Anzalone, Fabio Bucchieri, Silvestro Ennio D'Anna, Fabio L. M. Ricciardolo, Paola Brun, Bruno Balbi, Mauro Carone, Giovanni Zummo, Everly Conway de Macario, Alberto J. L. Macario, Antonino Di Stefano

**Affiliations:** 1 Dipartimento di Biomedicina Sperimentale e Neuroscienze Cliniche, Sezione di Anatomia Umana, Università degli Studi di Palermo, Palermo, Italy; 2 Istituto Euro-Mediterraneo di Scienza e Tecnologia (IEMEST), Palermo, Italy; 3 Dipartimento di Medicina Clinica e Sperimentale, Sezione di Malattie dell'Apparato Respiratorio, Università di Ferrara, Ferrara, Italy; 4 Fondazione S. Maugeri, IRCCS, Veruno (NO), Tradate (VA) and Cassano (BA), Italy; 5 Dipartimento di Scienze Cliniche e Biologiche, Università di Torino, Torino, Italy; 6 Dipartimento di Istologia, Microbiologia e Biotecnologie Mediche, Università di Padova, Padova, Italy; 7 Department of Microbiology and Immunology, School of Medicine, University of Maryland at Baltimore and Institute of Marine and Environmental Science (IMET), Baltimore, Maryland, United States of America; National Jewish Health, United States of America

## Abstract

**Background:**

It is increasingly clear that some heat shock proteins (Hsps) play a role in inflammation. Here, we report results showing participation of Hsp60 in the pathogenesis of chronic obstructive pulmonary diseases (COPD), as indicated by data from both *in vivo* and *in vitro* analyses.

**Methods and Results:**

Bronchial biopsies from patients with stable COPD, smoker controls with normal lung function, and non-smoker controls were studied. We quantified by immunohistochemistry levels of Hsp10, Hsp27, Hsp40, Hsp60, Hsp70, Hsp90, and HSF-1, along with levels of inflammatory markers. Hsp10, Hsp40, and Hsp60 were increased during progression of disease. We found also a positive correlation between the number of neutrophils and Hsp60 levels. Double-immunostaining showed that Hsp60-positive neutrophils were significantly increased in COPD patients. We then investigated *in vitro* the effect on Hsp60 expression in bronchial epithelial cells (16HBE) caused by oxidative stress, a hallmark of COPD mucosa, which we induced with H_2_O_2_. This stressor determined increased levels of Hsp60 through a gene up-regulation mechanism involving NFkB-p65. Release of Hsp60 in the extracellular medium by the bronchial epithelial cells was also increased after H_2_O_2_ treatment in the absence of cell death.

**Conclusions:**

This is the first report clearly pointing to participation of Hsps, particularly Hsp60, in COPD pathogenesis. Hsp60 induction by NFkB-p65 and its release by epithelial cells after oxidative stress can have a role in maintaining inflammation, e.g., by stimulating neutrophils activity. The data open new scenarios that might help in designing efficacious anti-inflammatory therapies centered on Hsp60 and applicable to COPD.

## Introduction

Chronic obstructive pulmonary disease (COPD) is a leading cause of morbidity and mortality worldwide [1]. It is characterized by airflow limitation that is not fully reversible, usually progressive and associated with an abnormal inflammatory response of the lung to noxious particles or gases [1]. Inflammation in COPD occurs in the central and peripheral airways as well as in lung parenchyma [2]. Management of patients with COPD is directed to maintain a stable condition, avoiding exacerbation episodes. However, a chronic inflammatory status in airways of stable COPD patients is present and is characterised by an increased number of CD8 lymphocytes, macrophages, and neutrophils [2].

Heat shock proteins (Hsps)-chaperones (hereinafter Hsps) are paradoxical molecules with beneficial, protective roles intracellularly but with potentially pathogenetic effects as they can initiate/perpetuate inflammation when secreted outside cells [3]. Intracellular Hsps have predominantly a cytoprotective effect in the lung [4]–[6]. By contrast, extracellular Hsps are signal molecules for the immune system, modulating the secretion of pro-inflammatory cytokines [7]–[11]. Although changes in the levels of Hsp60 and Hsp10 have been reported during bronchial carcinogenesis [12], [13], one of the most severe complications for COPD patients, participation of Hsps in COPD pathogenesis and progression has not, to our knowledge, been examined in any detail. For these reasons, we investigated in the bronchial mucosa the presence and levels of various Hsps and a pertinent transcription factor (i.e., heat shock factor-1, HSF-1), in relation to the COPD status.

Bronchial biopsies obtained from patients with mild/moderate and severe/very severe stable COPD, and control groups of either healthy smokers with normal lung function or non-smoking subjects, were studied applying a battery of complementary methods and experimental approaches. The *in vivo* results led us to focus on the mechanism of Hsp60 induction by oxidative stress, a hallmark of COPD mucosa, using *in vitro* experiments. All together, our results suggest a direct involvement of Hsp60 in COPD pathogenesis.

## Results

### Clinical characteristics of subjects studied

We obtained and studied bronchial biopsies from 55 Caucasian subjects: 32 had a diagnosis of COPD in a stable clinical state [1], [14], 12 were current or ex smokers with normal lung function, and 11 were non-smokers with normal lung function (Table 1). COPD patients were divided in two groups, according to their clinical stage (stage I–II: mild/moderate; or stage III–IV: severe/very severe; n = 14 and n = 18, respectively) [1]. Subjects in all groups were age-matched. The smoking history was similar in the three smoker groups: mild/moderate and severe/very severe COPD, and healthy smokers with normal lung function. Values of FEV_1_ (% predicted) and FEV_1_/FVC (%) were significantly different in the groups with mild/moderate and severe/very severe COPD compared to both control groups (healthy smokers and healthy non-smokers). Severe/very severe COPD patients also differed significantly from mild/moderate COPD patients (for overall groups, ANOVA test: p<0.0001 for FEV_1_% predicted and FEV_1_/FVC% values). Forty-one percent (n = 13) of the total COPD patients and 33% (n = 4) of healthy smokers with normal lung function also had symptoms of chronic bronchitis. There was no significant difference when COPD patients and healthy smokers were compared for the presence of chronic bronchitis (χ^2^, p = 0.658).

**Table 1:** Clinical characteristics of subjects studied.

Group	n	Age (years)	M/F	Smoking history (p-y)	Ex/current smokers	FEV_1_ preβ_2_ (% predicted)	FEV_1_ postβ_2_ (% predicted)	FEV_1_/FVC (%)
Control non-smokers	11	67± 10	10/1	0	0	116±14	ND	85±10
Control smokers with normal lung function	12	61±7	9/3	43±26	2/10	104±13	ND	81±6
Mild/moderate COPD	14	67±8	12/2	40±19	5/9	66±14^#^	72±12	60±8^#^
Severe/very severe COPD	18	66±9	11/7	54±36	13/5	35±8^#&^	38±9	44±10^#&^

Patients were classified according to GOLD (http://www-goldcopd.com) levels of severity for COPD into: mild/moderate (stages I–II) and severe/ very severe (stages III–IV).

Data are mean±SD. For COPD patients FEV_1_/FVC (%) are post-bronchodilator values.

Abbreviations: n, number of subjects; M, male; F, female, FEV_1_: forced expiratory volume in one second; FVC, forced vital capacity; ND, not determined; COPD, chronic obstructive pulmonary disease. Statistics. (ANOVA) #, p<0.0001, significantly different from control smokers with normal lung function and control never-smokers; ^&^, p<0.0001, significantly different from mild/moderate COPD.

### Measurement of inflammatory markers in the bronchial lamina propria showed increased levels of neutrophils, macrophages, and CD8 lymphocytes in COPD

We characterized and quantified several inflammatory markers in all specimens of airways mucosa obtained from stable COPD patients by immunohistochemistry. Measurements were made in the lamina propria of the mucosa. The results are summarized in Table 2. The number of neutrophils was significantly higher in the bronchial lamina propria of severe/very severe (median 173, range [47–500]) COPD patients compared with control smokers (101 [17–308], p = 0.011) and non-smokers (89 [59–179], p = 0.010). The number of myeloperoxidase (MPO) positive neutrophils was significantly higher in the lamina propria of severe/very severe (242 [129–387]) COPD patients than in the other three groups (p = 0.0009, p = 0.0016 and p = 0.047, in comparison with control non smokers, control healthy smokers and mild/moderate COPD, respectively). Compared with control non-smokers (284 [110–516]), the number of CD68 positive macrophages was significantly higher in severe/very severe (428 [204–1054], p = 0.033) and mild/moderate (566 [158–833], p = 0.036) COPD. Also the number of CD8 positive lymphocytes was significantly increased in severe/very severe (215 [59–355], p = 0.021) and mild/moderate (208 [86–523], p = 0.027) COPD compared to control non-smokers (120 [15–301]). The numbers of CD4- and FoxP3-positive cells did not differ significantly in the four groups of subjects. COPD patients with chronic bronchitis had a similar number of neutrophils and MPO-positive cells when compared with COPD patients without chronic bronchitis (not shown).

**Table 2:** Inflammatory markers in bronchial mucosa.

Target	Lamina propria (cells/mm^2^)				
	Control non-smokers	Control smokers with normal lung function	Mild/moderate COPD	Severe/very severe COPD	*p* value
CD4	168 (88–378)	218 (37–500)	245 (86–731)	252 (42–671)	0.360
CD8	120 (15–301)	187 (78–657)	208 (86–523) #	215 (59–355) #	0.125
CD68	284 (110–516)	369 (97–945)	566 (158–833) #	428 (204–1054) #	0.130
Neutrophil Elastase	89 (58–179)	101 (17–308)	125 (28–512)	173 (47–500) #ε	0.030
MPO	120 (54–250)	105 (43–258)	158 (64–306)	242 (129–387) #ε̂	0.003
FoxP3	86 (32–177)	105 (23–155)	92 (12–187)	86 (19–306)	0.990

Abbreviations: COPD, chronic obstructive pulmonary disease; MPO, myeloperoxidase; FoxP3: forkhead box P3. Data expressed as median (range). Statistics: Kruskal-Wallis test for multiple comparisons. For comparison between groups the Mann-Whitney U test was applied: #, p<0.05, significantly different from control non smokers; ε, p<0.05, significantly different from control smokers with normal lung function; ^, p<0.05, significantly different from mild/moderate COPD. The exact “p” values for comparison between groups are given in the Results section.

### Hsp10, Hsp40 and Hsp60 levels were increased in COPD

We quantified the levels of six Hsps and of HSF1 in bronchial mucosa of all subjects by immunohistochemistry. We quantified cell positivity separately in epithelium and in lamina propria (Table 3).

**Table 3:** Immunohistochemical quantification of Hsp and HSF-1 proteins in bronchial mucosa.

Protein in:	Control non-smokers	Control smokers with normal lung function	Mild/moderate COPD	Severe/very severe COPD	*p* value
**Bronchial epithelium (score 0-3)**					
Hsp10	1.0 (0.2–1.7)^c^	0.5 (0.2–2.5)	1.5 (0.2–3.0)	2.0 (0.5–2.7) #	0.031
Hsp27	2.0 (0.5–3.0)	2.0 (1.5–3.0)	2.7 (1.7–3.0)	2.5 (1.2–3.0)	0.164
Hsp40	1.5 (0.5–2.5)	1.5 (0.5–3.0)	2.5 0.5–2.7)	2.5 (0.5–3.0) #	0.112
Hsp60	0.7 (0.5–1.5)	1.2 (0.5–2.5)	1.7 (0.5–3.0)	1.5 (0.5–2.5) #	0.053
Hsp70	1.7 (0.2–2.7)	1.0 (0.2–2.7)	1.0 (0.2–2.8)	2.1 (0.2–2.8)	0.353
Hsp90	2.1 (0.5–2.7)	2.2 (0.5–2.7)	2.5 (1.0–3.0)	2.5 (0.5–3.0)	0.588
pHSF1	1.0 (0.5–2.5)	0.5 (0.5–1.5)	0.7(0.5–2.5)	0.5 (0.5–2.5)	0.056
**Lamina propria (cells/mm^2^)**					
Hsp10	97 (32–193)	126 (18–393)	233 (12–726) #	329 (68–639) #ε	0.009
Hsp27	387 (161–581)	353 (214–597)	452 (341–914)	460 (183–897)	0.111
Hsp40	145 (40–376)	191 (32–536)	277 (129–440) #ε	277 (54–753) #	0.032
Hsp60	113 (45–306)	115 (11–269)	161 (69–495)	193 (65–415) ε	0.120
Hsp70	103 (11–339)	91 (32–258)	97 (8–510)	161 (16–581)	0.356
Hsp90	188 (45–446)	241 (81–581)	328 (97–702)	266 (142–779)	0.384
pHSF1	77 (64–226)	64 (32–183)	139 (16–235)	69 (32–363)	0.267

Abbreviations: COPD, chronic obstructive pulmonary disease; MPO, myeloperoxidase. Data expressed as median (range). Statistics: The Kruskal-Wallis test was used for multiple comparisons followed by Mann-Whitney U test for comparison between groups: #, p<0.05, significantly different from control non smokers; ε, p<0.05, significantly different from control smokers with normal lung function; The exact “p” values for comparison between groups are given in the Results section.

**Figure 1:**
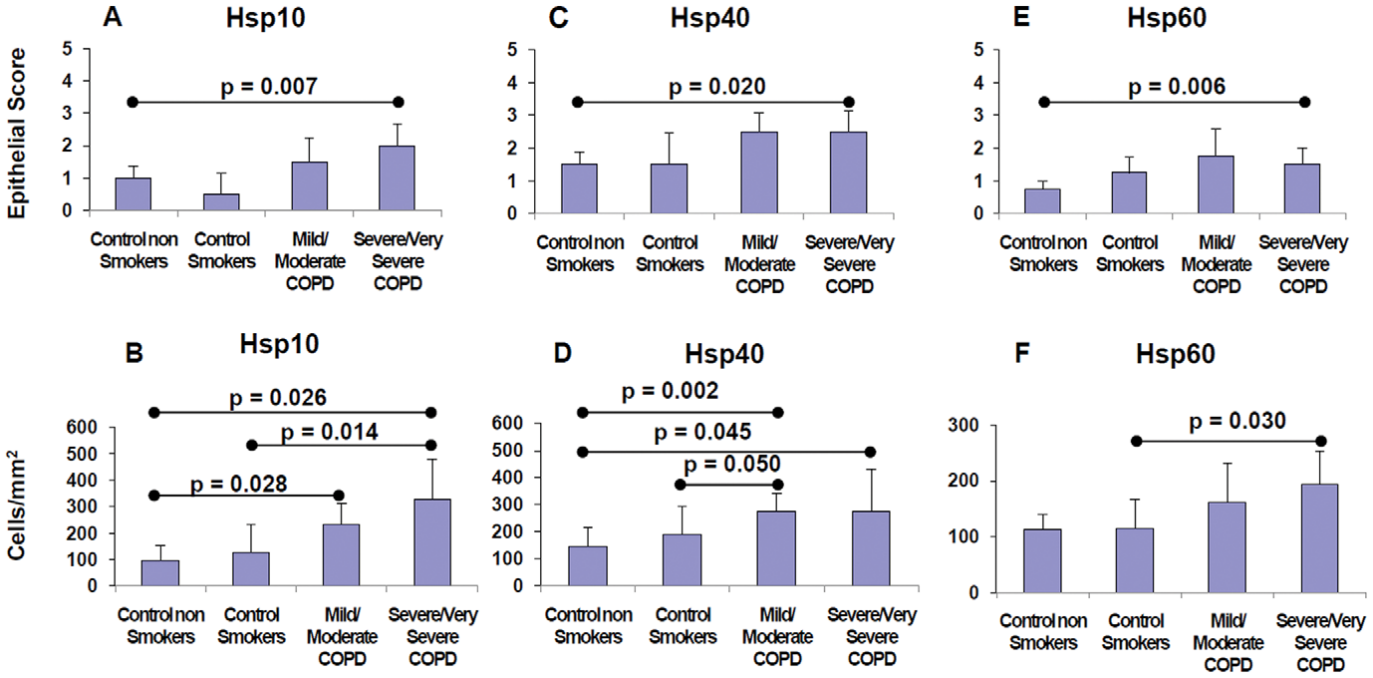
Measurement of Hsps in the bronchial epithelium and lamina propria: Comparison between groups of patients. Hsp10, Hsp40, and Hsp60 immunopositive cells in the epithelium (top panels A, C, E) and in the lamina propria (bottom panels B, D, F) of the four groups studied: Control non Smokers, Control Smokers, Mild/Moderate COPD, and Severe/Very Severe COPD. Results are expressed as the median and interquartile range (IQR) of scored (0–3, vertical axis) immunopositivity in the epithelium, or as number of immunopositive cells per square millimeter (vertical axis) in the lamina propria; “p” values are shown on top of the lines spanning the two groups being compared.

**Figure 2:**
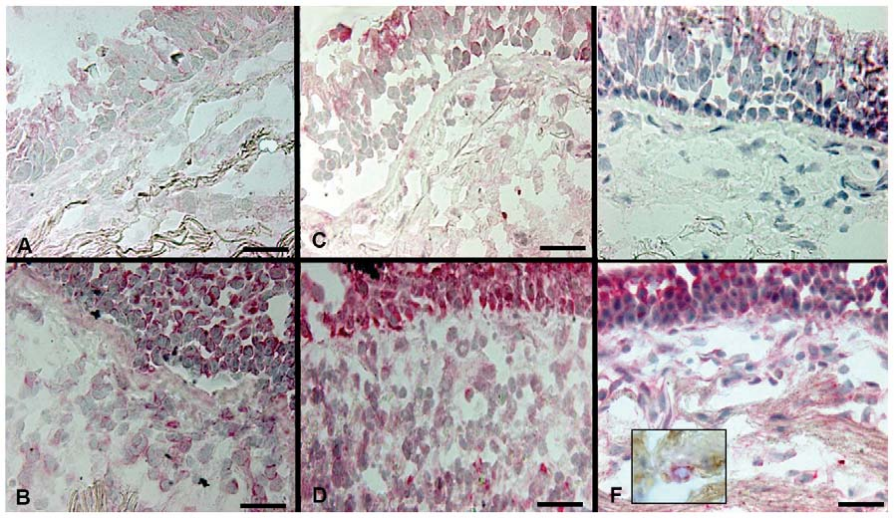
Hsps in the bronchial epithelium and lamina propria: Representative images. Photomicrographs showing frozen sections of bronchial mucosa from a control non smoker (A, C, E) and from a patient with severe stable COPD (B, D, F) immunostained to identify Hsp10 (A, B), Hsp40 (C, D), and Hsp60 (E, F). Nuclei were counterstained with haematoxylin (blue). Cells positive for Hsps are in red. Inset in F shows a cell double stained for neutrophil elastase (red) and Hsp60 (brown). Bar  = 50 microns.

All molecules were present in various percentages in epithelium and lamina propria of all groups. However, only Hsp10, Hsp40, and Hsp60 levels were increased in the bronchial epithelium of severe/very severe COPD compared to control non-smokers (Mann Whitney: p = 0.007, 0.020, and 0.006, respectively) (Figures 1 and 2). No significant differences were observed in the bronchial epithelium among the four groups of subjects for all other proteins investigated (not shown).

In lamina propria, a number of inflammatory cells, fibroblasts and smooth muscle cells were also positive for Hsps. Hsp27 was the most abundant Hsp observed in the bronchial lamina propria but without significant differences between the four groups of subjects. The number of Hsp60-positive cells was significantly higher in severe/very severe COPD compared to control smokers with normal lung function (Figure 1 and 2). The number of Hsp10 and Hsp40 positive cells was significantly increased in all stages of stable COPD compared to control smokers with normal lung function and non smoking subjects (Figure 1 and 2). No significant differences were observed in the bronchial lamina propria among the four groups of subjects for all other proteins investigated. COPD patients with chronic bronchitis had a similar number of Hsp10, Hsp40, and Hsp60 positive cells when compared with COPD patients without chronic bronchitis (not shown).

### RT-PCR in bronchial biopsies did not show differences in Hsp mRNA levels between the groups of patients

We performed a set of RT-PCR experiments to obtain clues about the mechanism, transcriptional or post-transcriptional gene regulation, responsible for Hsps increase in bronchial mucosa of COPD patients. Hsp10, Hsp40, and Hsp60 mRNAs levels did not differ significantly in the four groups of patients studied (Table 4).These results point to the occurrence of post-transcriptional events and/or the invasion of the mucosa by Hsps from other sources, as the cause of increased Hsp levels, *in vivo*.

**Table 4:** Hsp mRNA levels, expressed as the ratio [mRNA of gene of interest] / [mRNA of housekeeping gene (GAPDH)], in the bronchial mucosa.

Hsp	Control non-smokers (n = 7)	Control smokers with normal lung function (n = 8)	Mild/moderate COPD (n = 9)	Severe/very severe COPD (n = 9)	*p* value
Hsp10	0.19 (0.09–0.27)	0.11 (0.05–0.52)	0.15 (0.11–0.80)	0.32 (0.14–0.93)	0.414
Hsp40	0.96 (0.03–2.15)	1.08 (0.41–5.65)	0.94 (0.67–2.21)	0.86 (0.13–4.11)	0.872
Hsp60	1.0 (0.68–1.49)	0.96 (0.21–1.87)	1.3 (0.35–2.17)	1.31 (0.92–4.97)	0.134

Abbreviations: COPD, chronic obstructive pulmonary disease, Hsp, heat shock protein. Data are median (range). Relative levels of mRNAs are expressed as the ratio [mRNA gene of interest OD] / [mRNA housekeeping gene OD (GAPDH)]. Statistics: Kruskal-Wallis test was applied for multiple comparisons.

### Correlation analysis between Hsps protein levels, clinical characteristics, and inflammatory markers showed a correlation only between neutrophils and Hsp60

**Figure 3:**
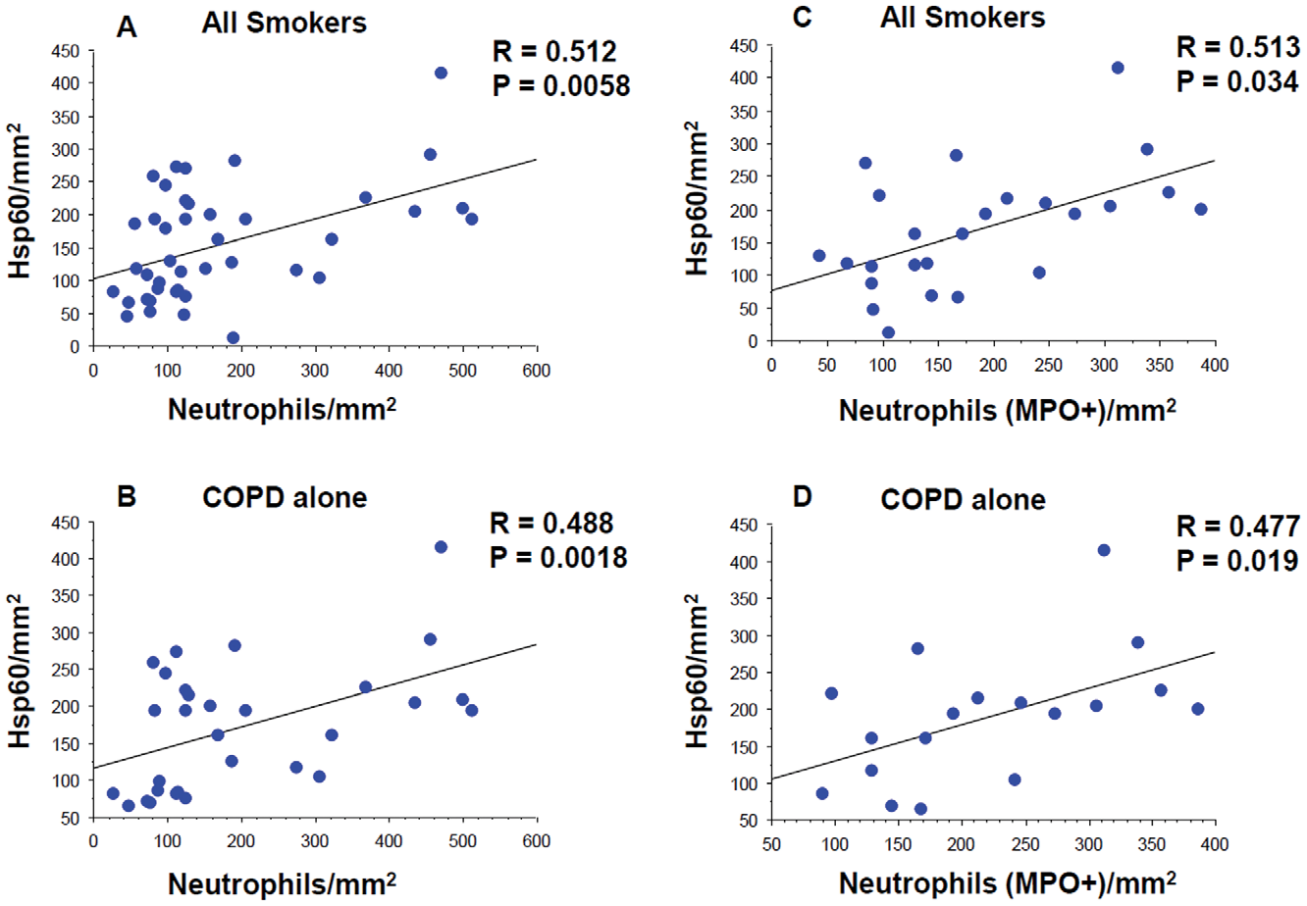
Correlations between neutrophils and Hsp60. Regression analysis between number of Hsp60 positive cells (vertical axis) and number of neutrophils (horizontal axis), panels A and B, and between number of Hsp60 positive cells (vertical axis) and number of MPO positive neutrophils (horizontal axis), panels C and D, in the lamina propria of all smokers (panels A and C) and of patients with COPD alone, considered as a single group (panels B and D).

We determined the correlation between Hsps and HSF-1 levels and either clinical characteristics or inflammatory markers. In all smokers, the number of Hsp60 positive cells in the lamina propria correlated positively and significantly with the number of neutrophils (r = 0.512, p = 0.0058) and with activated neutrophils expressing MPO (r = 0.513, p = 0.034) (Figure 3, panels A and C). Significant correlations were also observed when COPD patients were considered as a group (r = 0.488, p = 0.0018 and r = 0.477, p = 0.019 for neutrophils and MPO positive cells, respectively) (Figure 3, panels B and D). No other significant correlations were found between the other stress proteins investigated and any clinical parameter or inflammatory marker.

### Double immunohistochemical staining showed a significantly higher number of neutrophils expressing Hsp60 in COPD

The correlation between Hsp60 positive cells and neutrophils reported above let us to perform double-immunostaining experiments in four severe/very severe COPD patients and in four control healthy smokers. A number of neutrophils showed positivity for Hsp60 (Figure 2F, inset). In addition, the percentage of neutrophils expressing Hsp60 was significantly higher in severe/very severe stable COPD compared to control smokers with normal lung function [mean ± SD: 76 ± 7% vs 25 ± 14%, respectively; p<0.050].

### The *in vitro* Hsp60 expression and release in 16HBE bronchial epithelial cells were induced by a typical pulmonary stress pertinent to COPD

**Figure 4:**
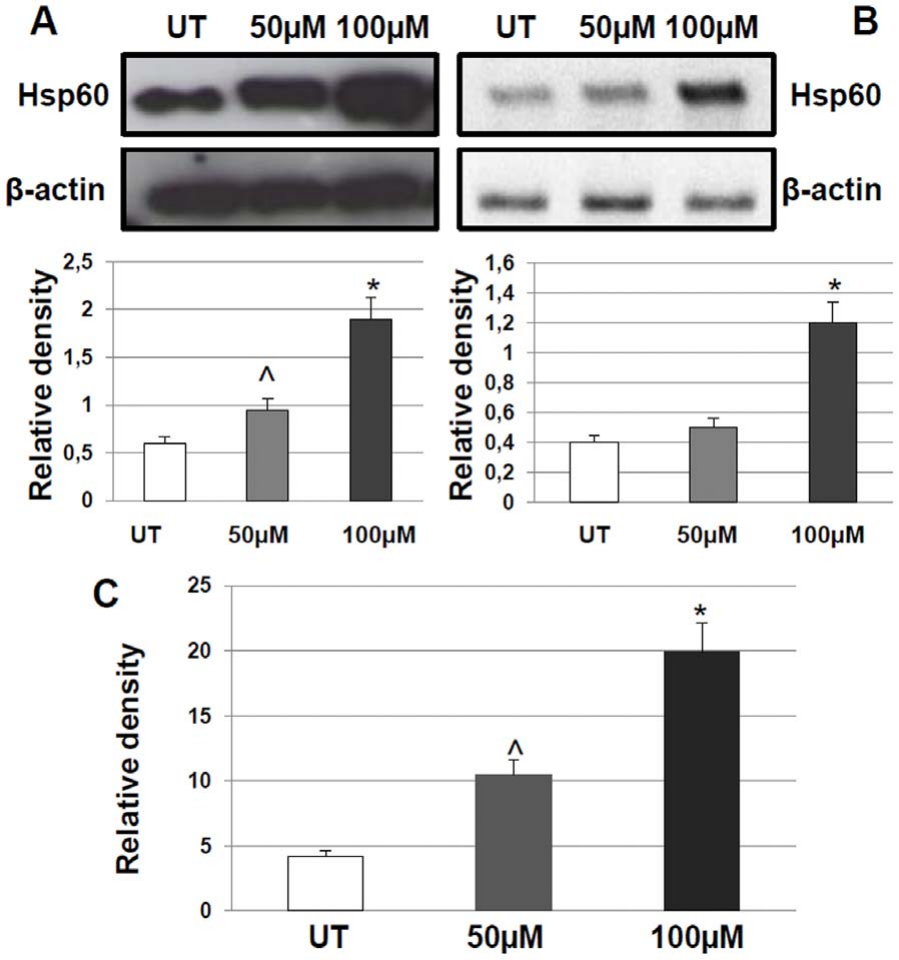
Impact of oxidative stress on the levels of Hsp60 in a human bronchial epithelial cell line. Panel A: Western blotting showing significantly increased levels of Hsp60 in 16HBE after treatment with 50 or 100 μM of H_2_O_2_ compared to untreated (UT) cells. The difference between 100 and 50 μM was also significant. Histograms represent the mean (SD) of the Hsp60/actin ratio. Panel B: RT-PCR showing significantly increased levels of Hsp60 only after treatment with 100 μM of H_2_O_2_ compared to UT treated cells. Panel C: Hsp60 increased levels in the extracellular medium (measured by ELISA tests) after treatment with 50 or 100 μM of H_2_O_2_ compared to untreated (UT) cells. ^: p<0.005; *: p<0.001.

We found of considerable interest the increase of Hsp60 in bronchial mucosa and the correlation of this increase with neutrophils in COPD patients. It is known that neutrophils are among the major players in inflammation in stable COPD patients [14]. Accumulation of Hsp60 in neutrophils raises the question: where does the Hsp60 come from? It is quite likely that neutrophils produce their own Hsp60. However, one cannot ignore that Hsp60 can reach neutrophils after release/secretion from other cells, as suggested by published data (see Discussion). Thus, we hypothesized that a proinflammatory stimulus, such as that due to oxidative stress, determines Hsp60 increase in epithelial cells as well as its release into the extracellular medium. To test this hypothesis we performed experiments *in vitro* using a human bronchial epithelial cell line (16HBE), which we had used before to study the effects of oxidative stress on Hsp levels [15]. The cells were treated with various concentrations of H_2_O_2_ and the effects on viability/apoptosis of various doses and exposure times were measured. The results indicated that treatments with 50µM and 100µM of H_2_O_2_ for 24 hours were not cytotoxic (viability at 50µM: 90.95+/−8.16%; at 100µM: 86.36+/−8.25%; p = 0.191 and p = 0.121, respectively). In cells treated with either dose, we found a significant increase of Hsp60 at both the protein and mRNA levels (Figure 4, A and B). The discrepancy between the Hsp60 mRNA results obtained *in vivo* (no detectable mRNA increase) and *in vitro* (significant increase) may be explained by the fact that for the former experiments we extracted mRNA from the whole mucosa whereas for the *in vitro* experiments we extracted the mRNA from epithelial cells only. ELISA tests showed that Hsp60 release by treated cells into the extracellular medium was significantly increased as compared with untreated cells (NT: 4.19 ng/ml; 50µM: 10.49 ng/ml; p<0.005; 100µM: 19.90 ng/ml; p<0.001) (Figure 4, C).

### NFkB-65 participated in the induction of the *hsp60* gene

**Figure 5:**
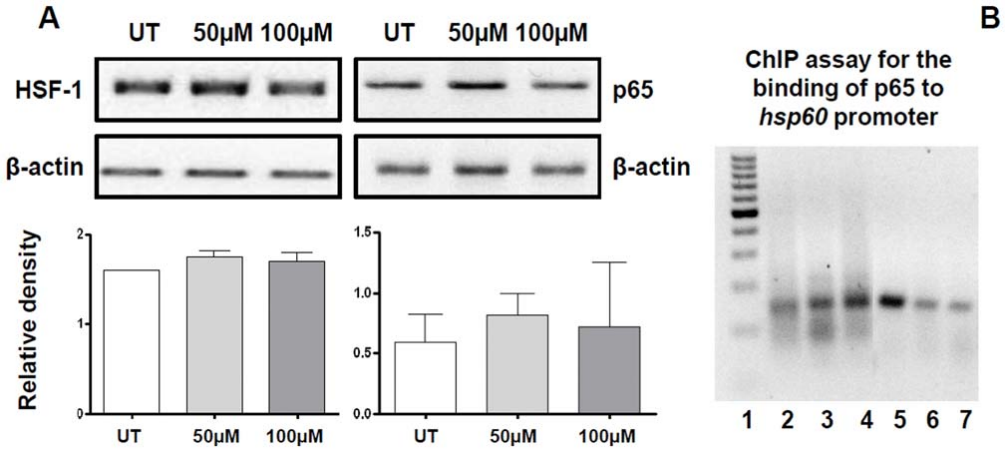
Quantification of mRNA in the bronchial biopsies and binding of NFkB-p65 to the human *hsp60*-gene promoter region. Panel A: Levels of HSF-1 and NFKB-p65 mRNA did not change significantly after treatment (50 or 100 µM of H_2_O_2_). Panel B: the binding of NFkB-p65 to the *hsp60* promoter was lower in untreated (UT) cells (lane 2) compared to the binding in cells treated with 50 or 100 µM of H_2_O_2_ (lanes 3 and 4, respectively). Lanes: 1, Marker; 5, DNA UT Positive control; 6, DNA UT Negative control; and 7, Input DNA.

To understand how treatment with H_2_O_2_ induces *hsp60* gene up-regulation in epithelial cells and whether HSF-1 and NFkB-p65, both considered pertinent Hsp transcription factors [16], are involved, we investigated the levels of mRNA for HSF-1 and NFkB-p65. They were present in all experimental conditions and no significant differences were found between the stressed and non-stressed cells (Figure 5, A). We then focused on NFkB-p65 because it has been reported that it is implicated in *hsp60* gene induction in heart cells [16]. Firstly, we searched by in *in silico* analysis putative binding sites for NFkB-p65 in the *hsp60* gene, and found three, one of which was on the promoter (Table 5). Subsequently we investigated by chromatin immunoprecipitation (ChIP) assay the binding of NFkB-p65 to the site on the promoter. The highest levels of binding were found in the gene from both, 50 and 100 µM of H_2_O_2_-treated cells, compared to untreated counterparts (Figure 5, B). Therefore, NFkB-p65 appears to be implicated in the up-regulation of the *hsp60* gene and, thus, lead to increased levels of Hsp60 in epithelial cells, which is accompanied by its release into the extracellular medium. Hence, the possibility exists for Hsp60 from epithelial cells of an inflamed airway mucosa to reach other cells, such as neutrophils.

**Table 5:** NFkB-p65 binding sites in the human *hsp60* gene.

Gene	Predicted NFkB-p65 binding site (nt)	Location
**Human ** ***hsp60*** **(Acc NC_000002)**	**1**) 342–356**2**) 3274–3286**3**) 12144–12156	PromoterIntronIntron
**Primer used for ChIP assay**		
**Site 1**	forward	reverse
**342-356**	5′-cgcagggtgtgcagattg-3′	5′-ggcgagtgagggacagagt-3′

## Discussion

Our study shows for the first time that in bronchial epithelium the levels of Hsp60 and its co-chaperonin Hsp10, and Hsp40 are significantly increased in patients with severe/very severe stable COPD compared to non-smokers with normal lung function. The same pattern was observed in lamina propria. Previous studies had indicated a crucial role of Hsp60 in modulating immunity and inflammation, particularly via the activation of macrophages and neutrophils. For example, Hsp60 activates human monocytes and macrophages by binding to CD14 with activation of p38MAPK [17], and stimulates monocytes to synthesize pro-inflammatory cytokines (such as TNFα, IL-12 and IL-15) and nitric oxide [18], synergizing with IFN-γ in inducing cytokine synthesis [19]. Extracellular Hsp60 can bind to the neutrophil's membrane, enhancing production of oxidants and release of proteases by it [19].

Hsp60 can induce regulatory T cells (Tregs) differentiation via the expression of FoxP3, the main transcription factor for Tregs in cord blood mononuclear cells [20]. Moreover, Hsp60 can also stimulate Tregs by both cell-to-cell contact and TLR2-mediated secretion of TGFβ and IL-10 [21], thus having anti-inflammatory function. Conflicting results have been reported on expression of FoxP3 in the airways of COPD patients [22], [23]. In our study, the levels of FoxP3 did not change significantly among groups and did not correlate with Hsp60. Thus, it is unlikely that Hsp60 has anti-inflammatory effects, at least through FoxP3 up-regulation, in stable COPD.

Neutrophil numbers can be elevated in the bronchial mucosa of stable COPD patients due to a variety of causes, including augmented chemotaxis, increased adhesion to collagens, and prolonged neutrophil survival [24]. The significant correlation we found between the number of Hsp60-positive cells and neutrophils in the bronchial mucosa of patients with stable COPD, suggests that this chaperonin plays a role in controlling neutrophil functions and bronchial inflammation in these patients. The Hsp60 molecules can be within and on the neutrophils and may originate in the neutrophils themselves and in other cells (e.g., epithelial) from which they gain the extracellular space and reach the neutrophils. Our experiments with a bronchial-epithelial cell line exposed to a pro-inflammatory stressor showed that Hsp60 expression increases during stress and is accompanied by its release into the extracellular medium (see later). Thus, the Hsp60, which augments in neutrophils of COPD mucosa, can be produced by the neutrophils themselves or captured by them from the extracellular space. The two mechanisms are possible and not mutually exclusive.

Oxidative stress, a hallmark of COPD mucosa [25], is a potent Hsp inducer, and Hsps may protect cells from death caused by oxidative stress [26], [27]. It is likely that the Hsp60 increase we found in neutrophils enhances their survival and activation, a situation that is likely to occur during bacterial infections often present in the bronchial mucosa of patients with stable COPD [28]. The possibility of infection will have to be investigated by examining sputum and bronchoalveolar-lavage samples taking before biopsy, samples that were not available for this study.

Our *in vivo* results led us to perform a set of *in vitro* experiments to determine whether an oxidant and pro-inflammatory stress (e.g., that caused by cell exposure to H_2_O_2_) induced Hsp60 up-regulation and release by bronchial epithelial cells (cell line16HBE). We found that: 1) Hsp60 levels in 16HBE increase after exposure to doses of H_2_O_2_ that do not affect cell viability; 2) The increase of Hsp60 levels is accompanied by release of the chaperonin in the extracellular medium; 3) Hsp60 increase is due at least in part to overexpression of the chaperonin gene, and in this up-regulation NFkB-p65 most likely plays a determinant role as indicated by its capacity to bind the chaperonin-gene promoter region. Furthermore, we have previously reported that NFkB-p65 is overexpressed in bronchial biopsies of COPD patients [29].

The results described above open new avenues for investigation, for example those aiming at clarifying which pathways (Golgi, exosomal, other) are involved in Hsp60 export from epithelial cells and whether Hsp60 secreted from bronchial epithelial cells may effectively cause activation of neutrophils.

With regard to the other Hsps that we found increased in bronchial biopsies, it is known that Hsp10 – the Hsp60 intramitochondrial co-chaperonin, also known as Early Pregnancy Factor [30] – has a potent immunosuppressive activity both *in vivo* and *in vitro* [31]–[33]. Bronchial inflammation in patients with stable COPD is characterized by the presence of activated Th1-Tc1 cells [2], [34]. We speculate that the Hsp10 increase observed in the bronchial mucosa could modulate and/or repress the Th1-Tc1 type of inflammatory response.

We reported previously that Hsp60 and Hsp10 levels decrease during carcinogenic steps in airways, i.e., from normal through dysplastic to neoplastic mucosa [12], [13]. COPD patients have a higher risk for developing bronchial cancer than other pneumopathies. Hence, our data suggest that monitoring Hsp60 and Hsp10 levels in patients with COPD could help in the identification of a subclass of patients (i.e., those in which Hsp60 and Hsp10 levels suddenly decrease) which would have a higher risk for cancer development.

The role of Hsp40 in bronchial inflammation is unclear. This molecule has traditionally been studied as an Hsp70 co-chaperone for its intracellular protein folding activity. In addition, Hsp40 up-regulation is considered a good marker for endoplasmic reticulum stress, particularly oxidative stress [35]. This information and our observations reported here suggest that elucidating the role of Hsp40 in the bronchial mucosa of stable COPD is promising research line.

Our results showed no significant differences for Hsp27, Hsp70, Hsp90, and pHSF-1 between stable COPD patients and controls. These data are at first glance in contrast with a previous study that demonstrated increased levels of these Hsps in the serum of patients with stable COPD as compared to controls [36]. In this previous study, however, no determinations were made on Hsps in bronchial mucosa specimens as reported here. Our data could, therefore, indicate that circulating Hsp27, Hsp70, and Hsp90, found by others in COPD patients [36], do not originate in the bronchial mucosa of greater bronchi but in other cells, e.g., peripheral airways or lung interstitial cells, or in other organs.

In conclusion, our data support the notion that Hsp60 can be involved in COPD pathogenesis. Hsp60 levels increase in the bronchial mucosa of patients with stable COPD and oxidative stress could be an inducer of this increment. Up-regulation of the *hsp60* gene by oxidative stress with participation of NFkB-p65 is probably responsible, at least in part, of the observed increase in Hsp60. Both, the release of Hsp60 from bronchial epithelial cells after an oxidant pro-inflammatory stimulus *in vitro* and the association we found in the bronchial mucosa between neutrophils and Hsp60 *in vivo*, favour the view that Hsp60 can be a marker of disease severity in COPD. However, only further studies with neutralization and/or administration of Hsp60 (first in animal models) will reveal the actual role of this molecule in the pathogenesis of COPD.

## Materials and Methods

### Subjects

All subjects were Caucasians and were recruited from the Respiratory Medicine Unit of the “Fondazione Salvatore Maugeri” (Veruno, Italy). The severity of the airflow obstruction was staged using current GOLD criteria [1]. All former smokers had stopped smoking for at least one year. COPD and chronic bronchitis were defined, according to international guidelines, as follows: COPD, presence of a post-bronchodilator forced expiratory volume in one second (FEV_1_)/forced vital capacity (FVC) ratio <70%; chronic bronchitis; presence of cough and sputum production for at least 3 months in each of two consecutive years [1]. All COPD patients were stable with no previous exacerbation in the six months before bronchoscopy. None of the subjects was treated with theophylline, antibiotics, antioxidants, mucolytics, and/or glucocorticoids in the month prior the bronchial biopsy. The study conformed to the Declaration of Helsinki and was approved by the ethic committee of the Fondazione Salvatore Maugeri [Veruno (Novara), Italy] (Approval number, up-dated on May 20, 2009, assigned by the local Technical and Scientific Committee: p81). Written informed consent was obtained from each subject and bronchial biopsies were performed according to the local ethic committee guidelines.

### Lung function tests and volumes

Pulmonary function tests were performed as previously described [14] according to published guidelines [37]. Pulmonary function tests included measurements of FEV_1_ and FEV_1_/FVC under baseline conditions in all the subjects examined (6200 Autobox Pulmonary Function Laboratory; Sensormedics Corp., Yorba Linda, CA). In order to assess the reversibility of airflow obstruction and post bronchodilator functional values the FEV_1_ and FEV_1_/FVC% measurements in the groups of subjects with FEV_1_/FVC%≤70% pre-bronchodilator was repeated 20 min after the inhalation of 0.4 mg of salbutamol.

### Fiberoptic bronchoscopy, collection and processing of bronchial biopsies

Subjects were at the bronchoscopy suite at 8.30 AM after having fasted from midnight and were pre-treated with atropine (0.6 mg IV) and midazolam (5–10 mg IV). Oxygen (3 l/min) was administered via nasal prongs throughout the procedure and oxygen saturation was monitored with a digital oximeter. Using local anaesthesia with lidocaine (4%) to the upper airways and larynx, a fiberoptic bronchoscope (Olympus BF10 Key-Med, Southend, UK) was passed through the nasal passages into the trachea. Further lidocaine (2%) was sprayed into the lower airways, and four bronchial biopsy specimens were taken from segmental and subsegmental airways of the right lower and upper lobes using size 19 cupped forceps. Bronchial biopsies for immunohistochemistry and RT-PCR were gently extracted from the forceps and processed for light microscopy as previously described [14]. Two samples were embedded in Tissue Tek II OCT (Miles Scientific, Naperville, IL), frozen within 15 min in isopentane pre-cooled in liquid nitrogen, and stored at –80°C. The best frozen sample was then oriented and 6μm thick cryostat sections were cut for immunohistochemical light microscopy analysis and 30μm thick cryostat sections were cut for RT-PCR and processed as described below.

### Immunohistochemistry

Two sections from each sample were stained applying immunohistochemical methods with a panel of antibodies specific for inflammatory markers or Hsps proteins (Table 6). Briefly, after blocking non-specific binding sites with serum derived from the same animal species as the secondary antibody, primary antibody was applied at optimal dilutions in TRIS-buffered saline (0.15 M saline containing 0.05 M TRIS-hydrochloric acid at pH 7.6) and incubated 1hr at room temperature in a humid chamber. Antibody binding was demonstrated with secondary antibodies anti mouse (Vector, BA 2000), anti rabbit (Vector, BA 1000) or anti goat (Vector, BA 5000) followed by ABC kit AP AK5000, Vectastain and fast-red substrate (red color). Slides were included in each staining run using human tonsil or nasal polyp as a positive control. For the negative control slides, normal goat, mouse or rabbit non-specific immunoglobulins (Santa Cruz Biotechnology, Santa Cruz, CA, USA) were used at the same protein concentration as the primary antibody.

**Table 6:** Primary antibodies and conditions used for identification of inflammatory markers, Hsps, and pHSF-1 protein.

Primary antibodies					
Target	Supplier	Catalog number	Source	Dilution	Positive control
CD4	Dako	M716	Mouse	1∶100	Human tonsil
CD8		M7103		1∶200	
CD68		M814		1∶200	
Neutrophil elastase		M752		1∶100	
Myeloperoxidase (MPO)		M748		1∶1000	
FoxP3	R&D	AF3240	Goat	1∶60	Nasal polyp
Hsp10	Santa Cruz Biotechnology, Inc.	Sc-20958	Rabbit	1∶100	Nasal polyp
Hsp27		Sc-1048	Goat	1∶200	
Hsp40		Sc-1801		1∶50	
Hsp60		Sc-1052		1∶100	
Hsp70		Sc-1060		1∶100	
Hsp90		Sc-1055		1∶150	
pHSF1		Sc-30443R	Rabbit	1∶300	

### Scoring system for immunohistochemistry

Morphometric measurements were performed with a light microscope (Leitz Biomed, Leica Cambridge, UK) connected to a video recorder linked to a computerized image system (Quantimet 500 Image Processing and Analysis System, Software Qwin V0200B, Leica). Light-microscopic analysis was performed at a magnification of 630x.

The immunostaining for all the antigens studied was scored (range: 0 =  absence of immunostaining to 3 =  extensive intense immunostaining) in the intact (columnar and basal epithelial cells) bronchial epithelium, as previously described [14]. The final result was expressed as the average of all scored fields performed in each biopsy. A mean±SD of 0.650±0.240 millimeters of epithelium was analyzed in COPD patients and control subjects.

Immunostained cells in lamina propria were quantified 100 μm beneath the epithelial basement membrane in several non-overlapping high-power fields until the whole specimen was examined. The final result, expressed as the number of positive cells per square millimeter, was calculated as the average of all the cellular counts performed in each biopsy.

### Double immunohistochemistry

Double immunostaining for identification of neutrophils expressing Hsp60 was performed as described previously [29]. Briefly, primary antibodies, goat anti-Hsp60, and mouse anti-neutrophil elastase (Table 6), were applied at the same dilutions as for single staining in TRIS-buffered saline (0.15 M saline containing 0.05 M TRIS-hydrochloric acid at pH 7.6) and incubated 1hr at room temperature in a humid chamber. Antibody binding was demonstrated with the use of secondary antibodies anti mouse (Vector, BA 2000) and anti goat (Vector, BA 5000) followed by ABC kit AP AK5000, Vectastain and fast-red substrate (red color) or ABC kit Elite PK6100, Vectastain, followed by Diaminobenzidine substrate (brown color), respectively. The immunostaining was scored as described above.

### Quantification of Hsp mRNA levels in bronchial biopsies

Quantification of Hsps mRNA was performed in 9 patients with severe/very severe COPD, 9 patients with mild/moderate COPD, 8 healthy smokers and 7 control non smokers randomly selected as representative of the larger groups analysed for the immunohistochemical study. Total RNA was extracted using the RNeasy Lipid Tissue Mini Kit (Qiagen, 74804, Milan, Italy) from 30-µm thick cryostat sections of bronchial biopsies following the manufacturers' instructions. Five nanograms of total RNA was used for cDNA synthesis, using the Enhanced Avian HS RT-PCR Kit (Sigma, HSRT100-1KT, Milan, Italy) and following the manufacturer's instructions. Primer pairs for Hsp10, Hsp40, and Hsp60 (Invitrogen, Milan, Italy) were as follows: Hsp10 (NM_002157) forward primer: cagtagtcgctgttggatcg; reverse primer: gcctccatattctgggagaag; Hsp40 (NM_006145) DNAJB1 forward primer: accagtggcccaggtaattt; reverse primer: ttgttccaactccccttcc; and Hsp60 (NM_002156) forward primer: gaagaaaaaggctggctgaa; reverse primer: tctccacagaaaggctgctt. Semiquantitative reverse transcriptase-polymerase chain reaction (RT-PCR) was carried out using the Enhanced Avian HS RT-PCR Kit (Sigma, HSRT100-1KT, Milan, Italy) following the manufacturer's protocol. The TotalLab TL120 program was used for quantification of amplified bands density. Relative levels of mRNAs were expressed as the ratio of optical density (OD) values of the gene of interest/ OD values of the housekeeping gene glyceraldehyde-3-phosphate dehydrogenase (GAPDH).

### Cell cultures and treatments

We used the SV40 large T antigen–transformed 16HBE cell line, a human bronchial epithelial cell line [38]. Cells were cultured in Dulbecco-modified Eagle's medium with 10% fetal calf serum (FCS) and supplemented with 2 mM glutamine, 50 U/ml penicillin, and 50 mg/streptomycin. Cells were grown as monolayers attached to the culture vessel and cultured at 37°C, 5% CO_2_, in a humidified incubator. Passage number of cells used in this study ranged from 12 to 35. Cell culture reagents were purchased from GIBCO BRL LIFE Technologies (Invitrogen, Italy). Prior to all the experiments, 70–80% cell monolayers were incubated in serum–free medium for the 24 hours (hrs). The cells were treated with different concentrations (0, 50 and 100µM) of H_2_O_2_ for 24hrs. All experiments were performed at least in triplicate.

### MTT Assay for 16HBE viability

MTT [3-(4,5 dimethylthiazol-2yl)-2,5-diphenyltetrazolium bromide] was obtained from Sigma (Italy). Briefly, 5x10^3^ 16HBE cells were plated in 200µl of complete medium per well in 96-well plates. Cells were treated as described in the previous paragraph and after 24hrs the used medium was replaced with new one containing MTT at a final concentration of 0.5mg/ml. After a 4-hr incubation period, cells were solubilized in 200µl of DMSO/well. Optical density (OD) was measured with a plate reader (Titertel Multiskan MCC/340, Flow Laboratories, Switzerland) at 570nm (630nm as reference). Cell viability was expressed as the percentage of OD value of treated cells compared with untreated controls, according to the following equation: Viability =  (OD Samples/ OD Control) x100.

### Cellular protein preparation and measurement from cell cultures

Untreated and treated cells were lysed into ice-cold lysis solution containing RIPA buffer, as previously described [38]. Lysates were then spun at 16,000×g for 30 min at 4°C, the supernatant was recovered, its protein concentration determined, and then stored at −80°C until use. Intracellular proteins were quantified with the Quant-iT™ protein assay kit (Invitrogen Molecular Probes, Italy), using the Qubit fluorometer according to the manufacturer's instructions (the kit is accurate for protein concentrations ranging from 12.5 µg/ml to 5 mg/ml).

### Western Blotting

Briefly, 40 µg of proteins was added to 4x Laemmli buffer and heated for 5 min at 95°C. Proteins were resolved by 12% SDS-PAGE along with a molecular weight marker (Bio-RAD Laboratories, Milan, Italy). Proteins were then transferred to nitrocellulose membranes. After transfer, all membranes were stained with Poinceau S to verify the quality of transfer and loading similarity. Anti-Hsp60 (LK1) monoclonal antibody was purchased from Sigma and used diluted 1∶1000; anti-β actin (W27) monoclonal antibody was purchased from Santa Cruz Biotechnologies (CA, USA) and used diluted 1∶1,000. Horseradish peroxidase-conjugated sheep anti-mouse antibody was purchased from Amersham Biosciences (Ge Healthcare, Italy). Before applying antibodies, the membranes were blocked with 5% not-fat milk, and probed for 12 hrs with the specific antibody, followed by incubation with horseradish peroxidase-conjugated second antibody. Blots were detected using the ECL (Amersham Bioscences) according to the manufacturer's instructions. Densitometric analysis of blots was performed using the NIH Image J 1.40 analysis program (National Institutes of Health, Bethesda, MD, USA).

### ELISA for Hsp60 level in cell culture supernatants of 16HBE cells

Quantitative comparison of Hsp60 levels in conditioned media cell culture supernatants was performed by ELISA, using commercial Hsp60 EIA kit from Stressgen Assay designs Inc (Ann Arbor, MI USA) as described [38]. The results were normalized for cell number and expressed as pg\ml\10^6^ cells.

### RNA extraction and quantification from 16HBE cells

The total RNA from untreated and treated cells was obtained using Charge Switch Total RNA Cell Kits (Invitrogen) and the quantity of RNA extracted was measured by Quant-IT RNA Assay kit using the Qubit fluorometer according to the manufacturer's instructions.

### RT-PCR with mRNA from 16HBE cells

RT-PCR was conducted with ImProm-II Reverse Transcriptase kit (Promega) and GoTaq Green Master Mix kit (Promega) according to the manufacturer's instructions. We detected the expression of Hsp60, HSF-1, and NFkB-p65 mRNAs using the following primers: Hsp60 forward primer gttccgagagctgaatgagg, reverse primer ctgagtcaggcccttctgtc; HSF1 forward primer tgctgtttgagctgggaga, reverse primer cgtttgtcctactggggcta; and NFkB-p65 forward primer gaccttttcaacttggcttcc, reverse primer tgatgctgtggtcagaagga. mRNA was normalized using a housekeeping gene (beta-actin) for each experimental condition. RT-PCR products were separated on 1% agarose gel.

### Chromatin immunoprecipitation (ChIP) assay

We first identified three NFkB-p65 binding sites in the human *hsp60* gene by sequence analysis with MatlNspector (version 7.7.3, Genomatix, http://www.genomatix.de/) (Table 5). Chromatin immunoprecipitation experiments were then performed to determine if NFkB-p65 would bind to the site identified in the promoter (Table 5). We used the ChIP IT kit (Active Motif) according to the manufacturer's instructions, as described by Wang et al. [16]. Approximately 1.5×10^7^ cells (either untreated or after treatment with H_2_O_2_) were fixed with formaldehyde (1% final concentration) for 10min. Cross-linking was terminated by the addition of glycine (1X final concentration). The chromatin was then cut into 150-300-450 bp fragments using digestion enzymes for 15 min. The chromatin was pre-cleared by adding Protein G beads to reduce non-specific background. Then 10µl of the pre-cleared chromatin was aliquoted as input. The rest of the pre-cleared chromatin of each sample was separated into three groups by adding specific antibody (NFkB-p65, C-20, Santa Cruz), positive control (RNApol II), or negative control (IgG). The immunoprecipitated DNA was eluted with ChIP elution buffer. The cross-linking was reversed with 5M NaCl and RNA was removed with RNase A, followed by digestion with proteinase K. The DNA was purified and PCR analysis was performed using the primers described in Table 5 and the products separated on a 3% agarose gel. PCR using the GAPDH primers on DNA isolated with the RNA pol II antibody (positive control) generated more product than similar reactions performed on DNA isolated using IgG (negative control), as described by manufacturer's instructions. The results were evaluated semi-quantitatively measuring the band intensity.

### Statistical analysis

Group data were expressed as mean (standard deviation) for functional data or median (range) or interquartile range (IQR) for morphologic data. Differences between groups were analyzed using analysis of variance (ANOVA) for functional and *in vitro* data. The ANOVA test was followed by the unpaired t-test for comparison between groups. The Kruskal Wallis test applied for morphologic data was followed by the Mann-Whitney U test for comparison between groups. Correlation coefficients were calculated using the Spearman rank method. Probability values of p<0.05 were considered significant. Data analysis was performed using the Stat View SE Graphics program (Abacus Concepts Inc., Berkeley, CA-USA).
